# Obituary

**Published:** 1855-09

**Authors:** 


*Obituary.*—Died of consumption, at sea, on the passage from New York to Liverpool, Charles Ap A. Bowen, M. D., Assistant Surgeon in the British Army, and lately Professor of Anatomy in the Geneva Medical College, aged twenty-three years.

Thus brief and imperfect is the announcement of the early death of one who had many friends among those who knew him, and whose professional anticipations were more than usually brilliant. Born in England, Dr. Bowen's parents removed, when he was a child, to Canada. He passed his medical pupilage under Prof. Austin Flint, of this city, graduating in the spring of 1853, at the University of Buffalo. Immediately on receiving his degree he accepted the position of Demonstrator of Anatomy in the Geneva Medical College. On the reorganization of the faculty of that school, in 1854, he was appointed Professor of Anatomy, a position which his zeal and attainment enabled him to fill with ability, though he was then but twenty-two years of age.

In May last, we were surprised to receive a letter from him announcing his resignation at Geneva, and his acceptance of the place of Assistant Surgeon in the British Army, to go to either the Baltic or Crimea, and offering at the same time to correspond with the Journal relative to medical matters at the seat of war. It was upon his voyage thither that his health, previously feeble, failed, and he was buried at sea.

In this the last letter we ever received from him he says, "I am about entering on a hard and dangerous service, and if I do not see you before I leave, the probability is that we shall never meet on 'terra firma' again."

The laughing, but half-serious prophecy is fulfilled; the hopes and ambitions of a warm, enthusiastic nature, are defeated by "the Conqueror Death." We will not write his eulogy, but those who read the record of his brief professional life — of two years only — will recognize the marks of an energetic and a manly nature, which would doubtless have secured a high distinction had time been given it.

